# IL-22 exacerbates weight loss in a murine model of chronic pulmonary *Pseudomonas aeruginosa* infection

**DOI:** 10.1016/j.jcf.2016.06.008

**Published:** 2016-11

**Authors:** Hannah K. Bayes, Neil D. Ritchie, Christopher Ward, Paul A. Corris, Malcolm Brodlie, Thomas J. Evans

**Affiliations:** a Institute of Infection, Immunity and Inflammation, University of Glasgow, G12 8TA, United Kingdom; b Institute of Cellular Medicine, Newcastle University, Framlington Place, Newcastle upon Tyne NE2 4HH, UK; c Institute of Transplantation, Freeman Hospital, Newcastle upon Tyne NE7 7DN, UK; d Paediatric Respiratory Medicine, Great North Children's Hospital, Queen Victoria Road, Newcastle upon Tyne NE1 4LP, UK

**Keywords:** PA, *Pseudomonas aeruginosa*, Cystic fibrosis, *Pseudomonas aeruginosa*, Chronic infection, Interleukin-22, Mucosal immunity

## Abstract

**Background:**

Interleukin (IL)-22 is a critical mediator of mucosal immunity and tissue regeneration, protecting against a number of respiratory pathogens. Whether IL-22 confers protection against chronic *Pseudomonas aeruginosa* (PA) infection in cystic fibrosis (CF) is unknown.

**Methods:**

Explanted CF lungs were examined for IL-22 production and immune-localization. A murine model of persistent pulmonary PA infection was used to examine production of IL-22 following infective challenge. The role of IL-22 was examined using IL-22 knockout (KO) animals.

**Results:**

IL-22 is produced within the adult CF lung and localizes to the airway epithelium. IL-22 is produced by murine pulmonary lymph node cells following lung infection. The absence of IL-22 resulted in no significant difference in acute mortality, bacterial burden, chronic infection rates, histological changes or neutrophilic inflammation in the chronic PA infection model. However, IL-22 KO animals lost less weight following infection.

**Conclusion:**

IL-22 is produced in the CF lung and in response to PA infection yet is dispensable in protection against chronic pulmonary *P. aeruginosa* infection in a murine model. However, we identified a novel role for the cytokine in promoting infection-related weight-loss, a significant prognostic factor in the CF population.

## 1 Introduction

*Pseudomonas aeruginosa* (PA) remains an important pathogen in cystic fibrosis (CF) with its extensive armament of virulence factors and evolving resistance profile [1], [2]. Chronic infections with mucoid PA strains develop in the majority of patients and result in poorer lung function and patient survival [1], [2], [3]. Current treatment strategies can delay onset of such chronic airways infection, but truly preventative measures, including anti-pseudomonal vaccines [4], remain elusive. Thus, identifying a critical anti-pseudomonal host response could enable therapeutic immunomodulation.

Interleukin-22 (IL-22), a member of the IL-10 family of cytokines, has recently attracted attention as a critical mediator of mucosal host defense, including the lung [5], [6]. IL-22 is produced by T helper (Th) 17 cells, Th22 cells, γδ T cells, natural killer (NK) T cells and innate lymphoid cells (ILCs) [5], [6]. Via its actions on non-haemopoieitic cells, IL-22 predominantly functions in tissue protection and repair—increasing innate defenses via expression of β-defensins, S100 proteins and lipocalin-2, maintaining transepithelial barrier functions and enhancing tissue regeneration [6]; thus representing an attractive therapeutic target. However, IL-22 may also exacerbate pro-inflammatory tissue-damaging responses in the lung when acting in synergy with the pro-inflammatory cytokine IL-17 [5], [7].

In respiratory infections, IL-22 provides critical immunity against *Klebsiella pneumoniae* [8], *Streptococcus pneumoniae* [9], *Chlamydia muridarum* [10], influenza [11], [12], as well as the CF pathogen *Aspergillus fumigatus* [13]. A protective role of IL-22 against pulmonary *P. aeruginosa* infection potentially exists [14], [15], but is yet to be clearly defined.

Interleukin-22 production is evident in patients with CF and pseudomonas infection. We have demonstrated that CF patients have peripheral Pseudomonas-specific Th22 and Th17 cells which may produce IL-22 in an antigen-specific manner and home to the infected lung [16]. In addition, explanted pulmonary lymph nodes of CF patients contain CD4^+^ IL-22^+^ T cells specific for *P. aeruginosa* antigens [17]. IL-22 transcript expression has also been found to be increased in lung tissue [17] as well as IL-22 levels in sputum and plasma [18] of CF patients.

We examine explanted CF lung airway IL-22 expression and IL-22 immune localization to further demonstrate cytokine involvement in the CF lung environment. To define the role of IL-22 in immunity to chronic *P. aeruginosa* infection, we utilized the agar bead model of persistent pulmonary infection [19] in mice, determining whether the cytokine is produced in response to infection and its role in determining acute outcome, chronic infection, inflammatory response, and infection-related weight loss.

## 2 Methods

### 2.1 Ethics

Approval was obtained from the Newcastle and North Tyneside 2 Research Ethics Committee (07/Q0906/47). Informed consent was obtained from all participants at the time of acceptance onto the active lung transplantation list at the Freeman Hospital, Newcastle Upon Tyne, UK. Characteristics of the 14 patients in the study who underwent lung transplantation for end-stage CF are contained in Supplementary Table 1.

### 2.2 Airway lavage samples

Airway lavage fluid was obtained from the explanted lungs of CF patients undergoing lung transplantation, as previously described [20].

IL-22 levels were measured via ELISA (eBioscience) with a lower limit of detection of 2 pg/mL.

### 2.3 Immunohistochemistry

Airway blocks were dissected to provide intermediate/large airways of 1–5 mm diameter with intact columnar airway epithelia. Formalin-fixed blocks were embedded in paraffin, sectioned at 5 μm and stained with haematoxylin and eosin to check for the presence of appropriate airway epithelia.

Sections for IL-22 staining were de-waxed in xylene and rehydrated through graded alcohols. Endogenous peroxidase activity was blocked by soaking sections in 0.3% methanolic hydrogen peroxide. The sections were washed for 10 min in running tap water and rinsed in tris-buffered saline (TBS). Antigen retrieval was performed in tris-EDTA-Triton X buffer at pH 9 (10 mM tris, 1 mM EDTA, 0.02% Triton X-100) at boiling point for 10 min. The sections were then allowed to cool for 20 min before blocking with 5% non-fat milk protein in TBS for 10 min. The primary antibody was a rabbit polyclonal immunoglobulin anti-IL-22 (Millipore, reference 06-1076) diluted 1:100 in 3% bovine serum albumin and applied for 24 h in a bioassay incubating tray at 4 °C.

Sections were washed twice with TBS and treated with a biotinylated horse anti-rabbit Envision secondary antibody system (Dako Laboratories) for 30 min. Sections were washed twice with TBS and treated with the ABC Vectastain Elite kit and DAB as per the manufacturer's instructions. The sections were finally counterstained with Carazzi's stain for 1 min and mounted. Negative controls were performed by omission of the primary antibody and treatment with isotype normal rabbit immunoglobulin (Dako Laboratories).

### 2.4 Agar bead infection model

The infection model was adapted from the protocol described by van Heeckeren et al. [19] and is widely utilized in CF research with inflammatory, microbial and remodeling akin to human CF lung disease [21], [22]. Wild-type (WT) mice were inoculated with sterile agar beads compared with *P. aeruginosa*-laden beads. In separate experiments examining the effect of IL-22, both WT and IL-22 knockout (IL-22 KO) mice were infected with PA-laden agar beads.

*P. aeruginosa*-laden agar beads were prepared the day before inoculation, stored overnight at 4 °C, and a different bead preparation used for each experiment. PA-laden beads were stored on ice throughout the murine surgery. Following inoculation of PA-laden beads, the administrated inoculum was confirmed by homogenization and quantitative bacteriology. Sterile agar beads were stored at 4 °C, used for several experiments and confirmed as sterile before and after each use.

For inoculation of beads, mice were anaesthetized using isofluorane via nose cone and the trachea exposed and cannulated (22G intravenous cannulae; BD Biosciences) under aseptic conditions. WT mice were infected at inoculum 1 × 10^6^ CFU per animal for comparison of response against sterile bead-treated animals. An initial low-dose experiment, of 1 × 10^5^ CFU/50 μl per animal, was undertaken to judge potential toxicity in genetically modified animals, followed by three subsequent experiments utilizing 1 × 10^6^ CFU/50 μl per animal. Daily weights were used as a measure of disease progression and those with weight loss of greater than 20% of baseline weight were culled prior to the predefined experiment end-point. Experiments were terminated at 2 weeks post-inoculation via carbon dioxide asphyxiation.

Bronchoalveolar lavage (BAL), pulmonary lymph nodes and lung tissue were harvested under aseptic conditions. A total of 1 ml of sterile phosphate-buffered saline (PBS) was infused and aspirated into the airways three times for each BAL sample. Thoracic lymph node cells were obtained from the bilateral mediastinal lymph nodes and single tracheobronchial lymph node [23].

### 2.5 *P. aeruginosa* strains

The clinical NH57388A strain was provided by N. Hoffmann (University of Copenhagen), the strain possesses a mutation in *mucA* resulting in alginate hyper-production [24]. The mucoid YH5 strain and non-mucoid GRI-1 strains were obtained locally, from a patient with CF and with ventilator-associated pneumonia, respectively. PA strains were maintained in − 80 °C stocks until required. Both the NH57388A and YH5 strains were used to form PA-laden agar beads as described previously [19]. For production of heat-killed PA, each strain was grown to mid-log phase in Luria–Bertani (LB) broth (Invitrogen) and the bacterial concentration at OD600 readings between 0.4 and 0.6 were quantified by serial dilution and plating to enumerate colony-forming units (CFU) (GeneQuant Pro spectrophotometer, Amersham Biosciences). PA were heated at a known concentration in PBS to 95 °C for 10 min.

### 2.6 Animals

All mice were used between 12 and 16 weeks of age. IL-22 KO mice were supplied by Genentech [25]. All murine lines had a C57BL/6 background. C57BL/6 mice bred in-house were used as wild-type controls for knockout comparisons. Animal work was carried out under a Project Licence as required by UK Home Office regulations as well as scrutiny and approval by an Institutional Review board at the University of Glasgow.

### 2.7 Bacteriology

Lung tissue was mechanically homogenized in 1 ml PBS. BAL and lung homogenates were plated for quantitative bacteriology on LB agar and examined after 24 and 48 h of incubation. Colonies were confirmed to be PA by appearance, Gram stain (BD Biosciences) and oxidase testing (Sigma-Aldrich). Chronic infection was defined as recovery of mucoid PA from lung cultures at 2 weeks post-inoculation. Pulmonary bacterial burden was calculated from BAL and right lung homogenates.

### 2.8 Neutrophil quantification

Blood and BAL samples underwent red blood cell (RBC) lysis (red cell lysis buffer; Sigma-Aldrich) prior to staining with Gr-1 (Ly6G; RB6-8C5; BioLegend). CountBright Absolute Counting Beads (Invitrogen) were added prior to washing cells and used according to the manufacturer's instructions. Stained cells were analyzed using FACs Aria (BD Biosciences) and FlowJo software (Treestar).

### 2.9 Cytokine measurement

Murine IL-17A, IL-17F, IL-21, interferon-γ (IFN-γ), and IL-22 were quantified by ELISA (all eBioscience). Lower limits of detection were: IL-17A < 4 pg/ml, IL-17F < 15 pg/ml, IL-21 < 16 pg/ml, IFN-γ < 15 pg/ml and IL-22 < 8 pg/mL. Cytokine levels less than the lower limits of detection of the assay were assigned a value of zero.

### 2.10 Histology scoring

Inflation fixed murine lungs were embedded in paraffin, sectioned at 5 μm and stained with haematoxylin and eosin. Histology was scored for peribronchial and alveolar involvement using a scoring system adapted from that described by Dubin et al. [15]. Scoring of each lung section was performed blindly at × 10 magnification by two independent investigators with an overall score given following assessment of a randomly selected whole lung section.

### 2.11 Mediastinal lymph node stimulation

Mediastinal lymph nodes were passed through 80 μm nitex mesh and RBCs lysed to form a single cell suspension. Cells were either left unstimulated or stimulated with heat-killed PA at MOI30. Following 3 days of culture, 100 μl of supernatant was removed for cytokine quantification.

### 2.12 Statistics

Results are presented as medians or, for technical repeats, mean and standard error of the mean (SEM). Mann–Whitney test was used for non-parametric comparisons. For parametric testing, Student's 2-sample *t*-test was used. Proportions were compared via Fishers' exact test. Comparison of animal weight changes were analysed using a repeated-measures ANOVA. Survival data was analysed via log-rank (Mantel–Cox) test. Statistical analysis was undertaken using Prism Version 6.0 (GraphPad Software). A *p*-value of < 0.05 was considered significant.

## 3 Results

### 3.1 IL-22 production and airway epithelial localization in explanted CF lung

**Fig. 1:**
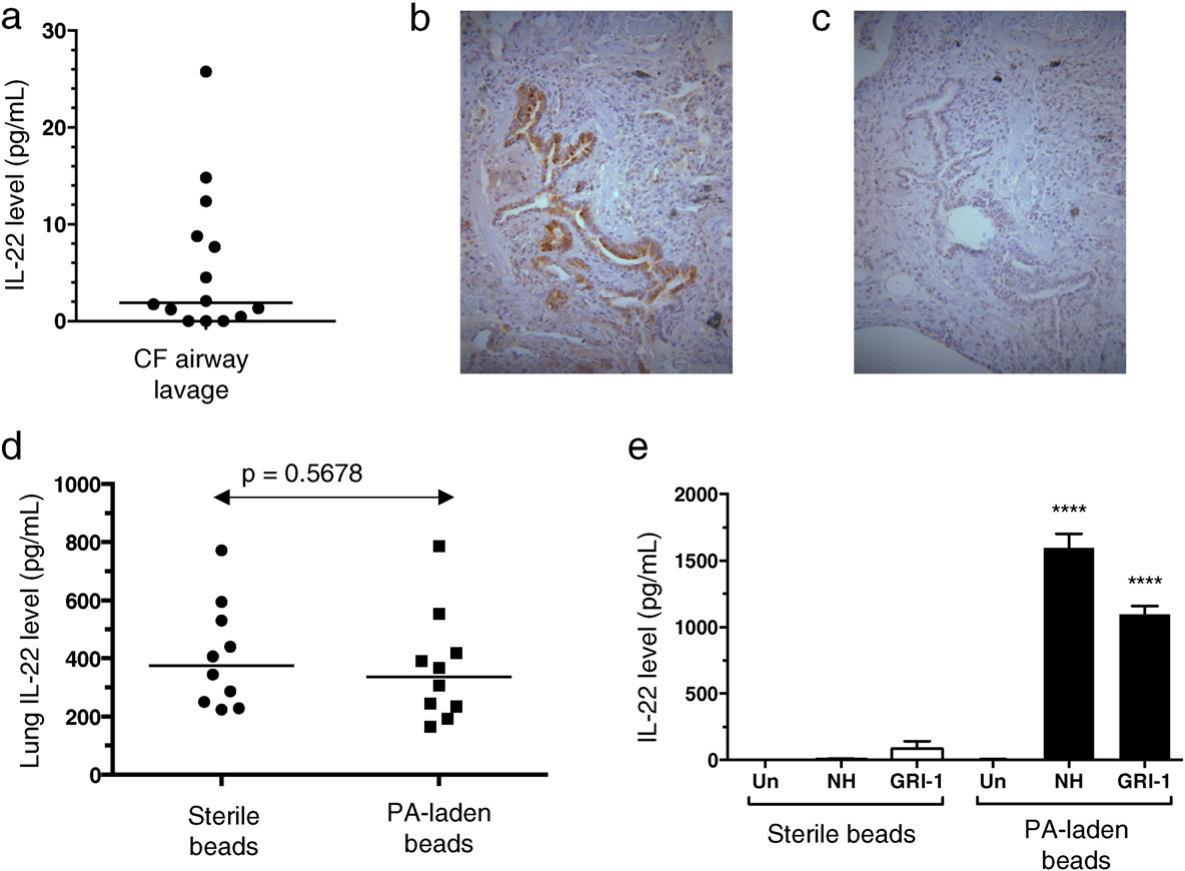
IL-22 responses in explanted human CF lung and murine persistent pulmonary *Pseudomonas aeruginosa* infection. (a) IL-22 levels were measured in airway lavage fluid from explanted lungs of CF patients undergoing lung transplantation. (b) Representative immunohistochemistry staining for IL-22 in the lower airway epithelium of an explanted lung from a person with CF undergoing transplantation, and (c) demonstrating corresponding isotype control (both × 20 magnification). (d-e) C57Bl6 mice were treated with intrapulmonary sterile agar beads or agar beads laden with *P. aeruginosa*, strain NH57388A. Lung homogenate (d) IL-22 levels measured at 2 weeks post-instillation. Data representative of 3 separate experiments. *P*-value related to Mann–Whitney test. (e) Two weeks after infection, mediastinal lymph node cells from mice treated as shown were stimulated *ex vivo* with heat-killed PA strains (NH57388A, denoted as NH, and GRI-1) at MOI 30 or left unstimulated (Un) for 3 days. Levels of IL-22 secretion are shown; bars are means and error bars are SEM. Un, unstimulated. NH, NH57388A. **** denotes *p* < 0.0001, comparison with corresponding sterile bead group via unpaired *t*-test. Representative of results from three separate experiments.

IL-22 was evident in airway lavage obtained from the explanted lungs of adult patients with CF undergoing transplantation (Fig. 1a). IL-22 was identified, via immunohistochemistry of explanted CF lung tissue, to localize to the airway epithelium with more sparse staining in lung parenchyma (Fig. 1b).

### 3.2 IL-22 production in PA-specific immune responses following infection

Comparison of animals treated with sterile or PA-laden agar beads demonstrated no significant difference detectable in IL-22 levels in BAL or lung homogenates at either 48 h (data not shown) or 2 weeks (Fig. 1d) post-instillation. In addition, there was no significant difference between infected animals and sterile bead controls in other pulmonary cytokine levels related to Th17 cells, i.e. IL-17A, IL-17F, and IL-21 (Supplementary Fig. 1). No detectable IL-17A or IL-22 was found in the BAL of treatment-naïve wild-type mice.

We thus sought to ascertain if PA-specific immune responses had been induced by the persistent pulmonary PA infection and were able to produce IL-22 in an antigen-specific manner. Two weeks following PA infection, there was a marked enlargement of the mediastinal lymph nodes compared to control animals (3.56 × 10^6^ cells mean value for NH57388A-infected animals versus 2.83 × 10^5^ cells mean value for sterile bead-treated animals; three separate experiments, *p* < 0.05). *Ex vivo* restimulation of these lymph node cells from infected animals with heat-killed bacteria of the infecting strain NH73788A or an unrelated clinical PA strain GRI-1 for 3 days resulted in robust production of IL-22, which was not evident in animals receiving sterile beads (Fig. 1e). Similarly *P. aeruginosa*-specific production of IL-17A by infected animals was evident (data not shown). This suggests that IL-22 production can occur as a specific response to pulmonary *Pseudomonas* infection and that there is migration to and/or expansion of PA-specific IL-22^+^ producing cells within the thoracic lymph nodes in response to pulmonary infection.

### 3.3 IL-22 absence does not alter acute responses to pulmonary PA infection

Having demonstrated that IL-22 could be produced locally in response to pulmonary *P. aeruginosa* infection, we compared the response to infection in wild-type mice and animals lacking the ability to produce IL-22. The response was assessed to two clinical mucoid strains of PA, NH57388A and YH5, including at low- and high-inoculation dose in the latter. To ensure IL-22 KO animals did not have altered responses to the agar beads per se, we examined the impact of sterile agar beads in IL-22 KO and WT mice at 48-h post-bead instillation. Importantly, we found no difference in weight loss, clinical score, microbiology (no organisms isolated) or blood and BAL neutrophil levels between IL-22 KO and WT animals treated with sterile beads (data not shown).

**Fig. 2:**
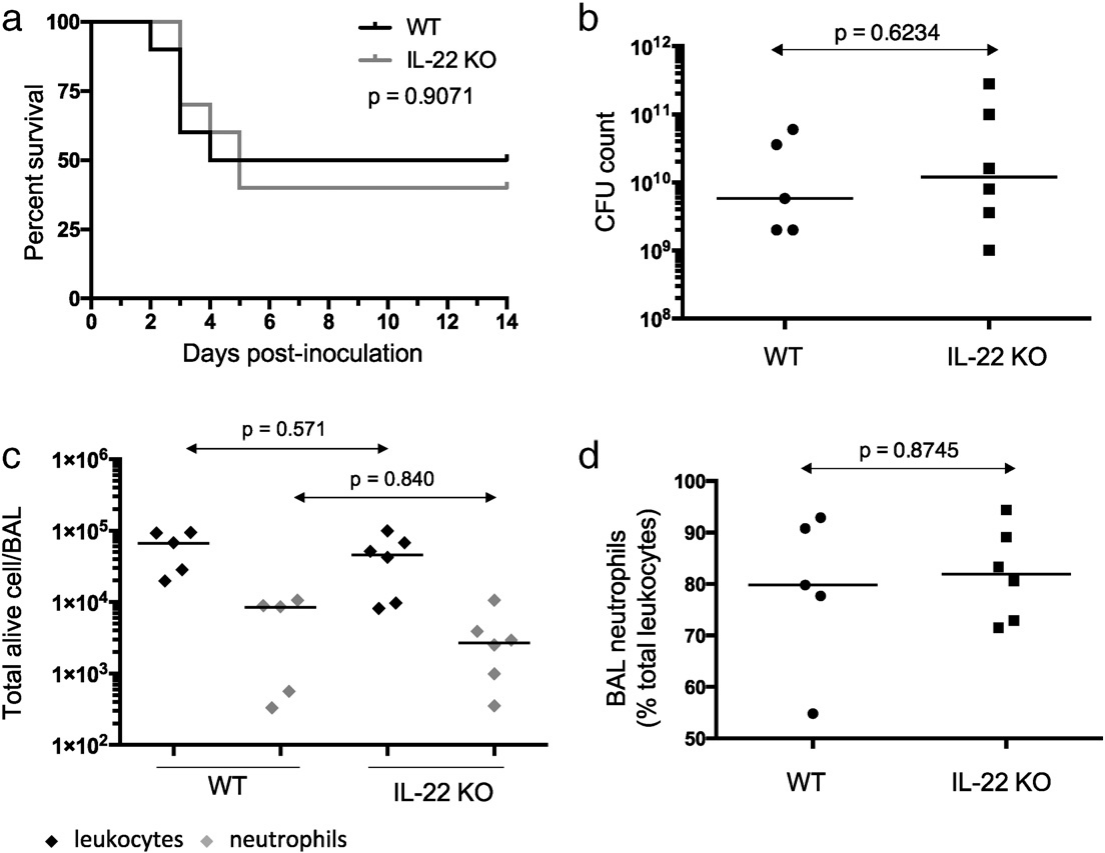
Acute response to pulmonary *Pseudomonas aeruginosa* infection with strain NH57388A in IL-22 knockout and wild-type mice. IL-22 knockout (IL-22 KO) (*N* = 10) and wild type (WT) (*N* = 10) mice received agar beads laden with PA strain NH57388A. a, Survival comparison between the two murine strains; *p*-value relates to comparison via log-rank (Mantel–Cox) test. In animals succumbing prior to termination of the experiment (early deaths; i.e. 3–5 days post-infection), pulmonary PA bacterial burden was measured on total lung homogenates and bronchoalveolar lavage (BAL) (b). c,d, BAL leukocytes were strained for Gr-1 followed by flow cytometry at the time of termination. c, absolute BAL total alive leukocyte and neutrophil counts. d, percentage BAL neutrophil counts. In b–d, line indicates the median and *p*-values relate to Mann–Whitney test.

IL-22 has previously been shown to play a critical role in protection from acute pulmonary infection with another Gram-negative organism by maintaining transepithelial resistance and preventing systemic spread [8], [13]. In contrast, there was no significant difference in acute mortality (i.e. prior to pre-defined experimental endpoint) in any of the experiments comparing persistent PA infection in IL-22 knockout and WT animals. Higher rates of acute mortality in WT and IL-22 KO groups were seen to the NH57388A strain compared with YH5 stain. Combined, there were a total of 7 acute deaths from 40 treated animals (17.5%) in both the presence and absence of IL-22. In both WT and IL-22 KO groups, early termination was due to weight loss greater than 20% of baseline and occurred over the same time period of 3–5 days post-inoculation (Fig. 2a). There was also no significant difference in bacterial burden (Fig. 2b) nor in the level of acute inflammation, measured via BAL leukocyte and neutrophil levels (Fig. 2c and d) in the lungs of IL-22 KO compared with WT animals culled at these early time-points (i.e. 3-5 days post-infection). Importantly, due to the role of IL-22 in transepithelial resistance, there was no bacterial growth on terminal blood cultures of any animal at any time-point. Thus, acute resistance to pulmonary PA infection was unaltered in the absence of IL-22.

### 3.4 IL-22 signaling does not provide resistance to chronic pulmonary PA infection

**Fig. 3:**
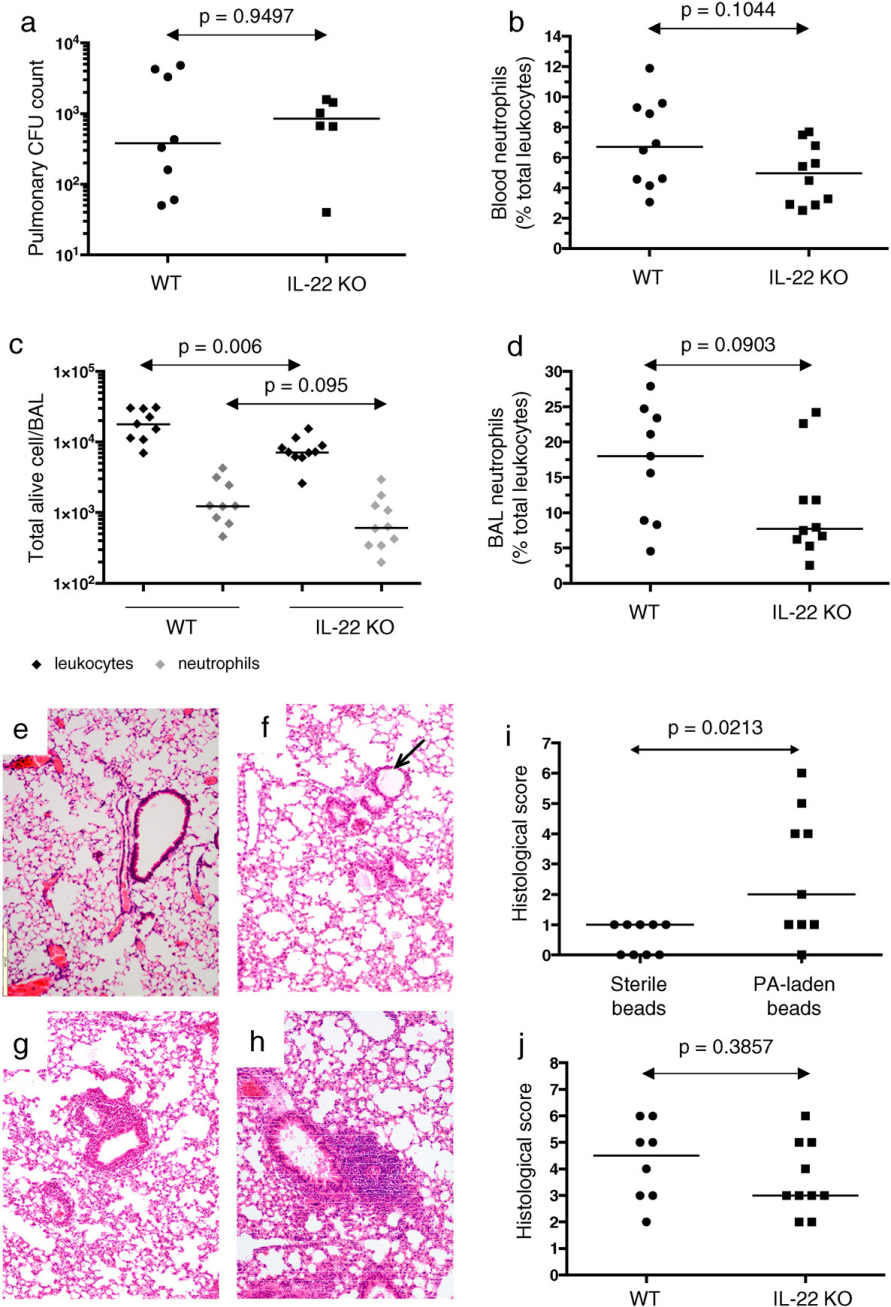
Bacterial burden and inflammatory response in IL-22 knockout and wild-type mice with chronic pulmonary *Pseudomonas aeruginosa* infection. IL-22 knockout (IL-22 KO) and wild type (WT) mice received intra-pulmonary agar beads laden with PA with pulmonary bacterial burden, neutrophil and histological response assessed at 2 weeks post-inoculation. (a) Pulmonary bacterial burden (CFU count, calculated from combination of bronchoalveolar lavage and right lung homogenate). Combined results for 3 experiments utilizing YH5 strain. (b-d), Blood (b) and BAL (c,d) neutrophil levels, measured via straining for Gr-1 and flow cytometry. Representative of results for three separate experiments. (c) Absolute BAL total alive leukocyte and neutrophil counts. (d) Percentage BAL neutrophil counts. (e–h) H&E staining of representative lung sections (all at × 10 magnification) in WT mice with no treatment (healthy control); (e) 2 weeks post-inoculation with sterile agar beads in WT mice (f) and 2 weeks post-inoculation with YH5-laden agar beads in WT (g) and IL-22 KO (h) mice. Small black arrow highlights agar bead within airway. (i) Quantitative histological scores of WT animals treated with sterile agar beads (*N* = 9) or PA-laden agar beads (*N* = 9). (j) Quantitative histological scores of WT (*N* = 8) and IL-22 KO (*N* = 10) animals treated with YH5-laden agar beads. Line denotes median score. *P*-values relate to Mann–Whitney tests.

At two weeks post-inoculation, there was no significant difference in chronic pulmonary *P. aeruginosa* infection rates between inoculated IL-22 KO and WT animals in any experiment. At low inoculum YH5 infection, 37.8% (3/8) WT compared with 14.3% (1/7) IL-22 knockouts were chronically infected (*p* = 0.5962, Fishers' exact test). In the high inoculum experiments, chronic infection was seen in 25% (5/20 animals) in both WT and IL-22 KO groups. Pulmonary bacterial burden in chronically infected animals was also similar in IL-22 knockout (median 845 CFU, IQR: 505–1468) and wild-type (median 380, IQR: 505–1468) animals (*p* = 0.9497, Fig. 3a). Thus, IL-22 does not provide critical protection against the development of persistent pulmonary PA infection.

### 3.5 IL-22 absence does not alter inflammatory responses to chronic PA infection

The absence of IL-22 signaling did not influence the level of chronic inflammation measured by neutrophil levels both in peripheral blood (Fig. 3b) and BAL (Fig. 3c and d). Total leukocytes were marginally less in IL-22 KO BAL (Fig. 3c) but there was no significant difference both for all animals inoculated with PA-laden agar beads (Fig. 3b–d) and for those remaining chronically infected (data not shown) in neutrophilic inflammation.

Persistent pulmonary inflammatory changes were evident 2 weeks post-inoculation of PA-laden agar beads (Fig. 3d-i). Minimal changes were seen in response to sterile agar bead instillation per se (Fig. 3e). Infected lungs demonstrated localized peribronchial monocytic infiltrates with an agar bead frequently evident in the adjacent airway (Fig. 3f) and higher histological score in WT animals treated with PA-laden compared with sterile agar beads (Fig. 3i). Histological changes were similar in WT (Fig. 3g) and IL-22 KO (Fig. 3h) mice treated with PA-laden agar beads, with no significant difference in lung histological scores at 2 weeks post-inoculation (Fig. 3j).

### 3.6 Weight loss related to infection is attenuated by the absence of IL-22

**Fig. 4:**
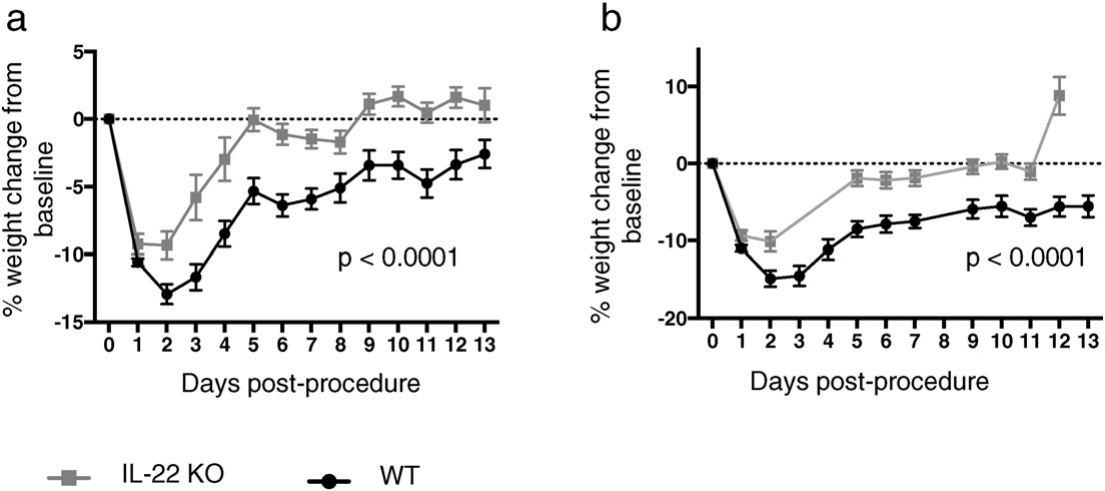
Weight loss in wild-type and IL-22 knockout mice treated with *Pseudomonas*-laden agar beads. Body weight measured daily in wild-type (WT) and IL-22 knockout (IL-22 KO) mice treated with *Pseudomonas aeruginosa*-laden agar beads. Percentage weight change from baseline for pooled experiments. (a) All animals infected with PA-laden beads (WT = 28, IL-22 KO = 27). (b) Only animals remaining chronically infected with PA at 2 weeks post-inoculation (WT = 8, IL-22 KO = 6). Each point represents mean of group and SEM. (a) Difference between groups significant at *p* < 0.0001 via repeated measures ANOVA. (b) Difference between groups significant at *p* < 0.0001 via repeated measures ANOVA.

Post-procedure weight loss was significantly greater and sustained in WT animals treated with PA-laden beads compared with sterile bead controls (*p* < 0.0001, via repeated measures ANOVA; data not shown). Interestingly, there was significantly less weight loss in IL-22 knockout animals compared with WT mice treated with PA-laden agar beads (Fig. 4). This lower level of weight loss in the absence of IL-22 remained evident when only chronically infected animals were compared (*p* < 0.0001, via repeated measures ANOVA; Fig. 4b).

## 4 Discussion

Consistent with our previous work demonstrating IL-22-producing *P. aeruginosa*-specific responses in the peripheral blood of patients with CF [16], we demonstrate airway IL-22 production and IL-22 localization to the airway epithelium in explanted lung tissue from CF patients. In addition, we demonstrate that murine persistent pulmonary PA infection elicits an antigen-specific response able to produce IL-22 locally in draining lymph nodes. We found no difference in IL-22, and other related cytokines, in the lungs of animals treated with PA compared with sterile bead-treated animals. Trauma itself may induce cytokine production and thus the observed lack of difference may be attributable to the mode of bead inoculation.

Despite its reported protective role against a range of pulmonary pathogens [8], [9], [10], [11], [12], we found no evidence that IL-22 is critical in preventing the establishment of chronic pulmonary *P. aeruginosa* infection. The most striking effect of IL-22 was in relation to infection-associated weight loss. Poor growth and impaired nutritional status are now recognized as important determinants of pulmonary health and survival in CF [3], [26]. Determinants of low weight are likely multifactorial; however, pulmonary PA infection and the associated inflammation are important contributors [27]. Our model demonstrated weight loss in relation to *P. aeruginosa* infection, which was significantly less marked in the absence of IL-22 (Fig. 4). The mechanism by which IL-22 causes weight loss in pulmonary PA infection is not clear and may include multiple direct and indirect effects of the cytokine. We found no difference in bacterial burden or pulmonary inflammation levels in the presence or absence of IL-22 that might explain the difference between groups. There may yet be a pathological effect of IL-22 in promoting systemic and airways inflammation, perhaps regulated by IL-17 [7], which was undetected in the present study but which exacerbates the weight loss associated with infection. In addition, recent data suggests a new role for IL-22 in regulating metabolic homeostasis including insulin sensitivity [28] and production [29] as well as regulation of lipid metabolism in liver and adipose [28]; with studies to-date identifying complex and beneficial effects on metabolism in murine models of obesity [28], [29]. Thus, our observed increased weight loss in the presence of IL-22 could also represent an effect on metabolism independent of the cytokine's immune function.

Interleukin-22 exerts an important role in maintaining mucosal integrity with resultant prevention of systemic spread of organisms [8]. Thus, importantly, we demonstrated IL-22 localization to the airway epithelium in human CF lung (Fig. 1), which may represent production and/or binding of the cytokine at this site of immune activity. However, akin to *P. aeruginosa* infection in patients, the experimentally persistent PA infection model demonstrates that intrabronchial sepsis and transepithelial invasion is not a significant component of pathogenesis. This may explain the lack of importance of IL-22 in chronic pulmonary infection observed here compared with the findings of others in acute lung infection where transepithelial spread significantly influences disease outcome [8], [9], [11], [14].

Interleukin-22 is implicated in mucosal immunity and homeostasis both in the lung and gut, and the inter-relationship of gut and lung immunity is an area of growing interest. Shih et al. demonstrate that mice lacking IL-22 and IL-22 neutralisation in mice via exogenous anti-IL-22 antibody resulted in altered gut microflora, with increased segmented filamentous bacteria (SFB) that can promote Th17 cell development [30]. Gauguet et al. demonstrated that the gut microbiome may influence pulmonary immunity as mice with gut SFB showing increased BAL IL-22 levels and decreased susceptibility to acute *S. aureus* pneumonia [31]. We did not examine the gut microflora of either the WT or KO animals used in our research and whether the gut microbiome has effects on persistent pulmonary infections is unexplored. However, consideration of gut–lung interactions in the context of CF would need to take account of the increasing evidence of altered gut microbiota in CF patients [32], [33].

A further function of IL-22 in mucosal immunity is in the production of anti-microbial peptides including β-defensins and S100 proteins [8]. Elevated levels of such anti-microbial proteins have been demonstrated in the airway secretions of patients with CF [34]. In addition, in the context of ocular infection, human β-defensin 2 plays an important role in defense against *P. aeruginosa* [35]. However, such anti-microbial factors may be induced by alternative non-IL-22 pathways, including via IL-17 [36], [37], and thus may further explain our observed lack of impact of IL-22 on bacterial burden or infection rates in persistent PA infection. Cross-talk between cytokines is undoubtedly important in immunopathological outcomes [5], [7] and thus dual manipulation of IL-22 and IL-17 cytokines may provide further insights into the important interactions within the complex cytokine milieu of the infected CF lung.

Our infection model utilized PA-laden agar beads instilled into animals with no previous lung infection. Interestingly, Mear et al. found that prior *Candida albicans* lung infection resulted in IL-22 production from ILCs that provided cross-protection against subsequent acute *P. aeruginosa* pneumonia [14]. Similarly IL-22 was found to protect against superimposed infection with *S. pneumoniae* following respiratory influenza A infection [11]. Thus, assessing whether a similar IL-22 response can be induced by an early CF pathogen, which provides cross-protection against subsequent chronic mucoid PA infection, still merits consideration.

Interleukin-22 functions in appropriate repair following pulmonary insults. Simonian et al. used an experimental model of pulmonary fibrosis, using repeated exposure to *Bacillus subtilis*, demonstrating that IL-22 blockade results in accelerated lung fibrosis [38]. A similar anti-fibrotic role for IL-22 has been demonstrated following influenza infection with a corresponding benefit in preserving lung function [12]. Whether IL-22 has a similar longer-term reparative role in the CF lung is of interest but cannot be answered in the present study. Our preliminary analysis of lung collagen deposition (via Picro-sirius red collagen lung staining) in response to 2 weeks of pulmonary PA infection suggested no difference between wild-type and IL-22 KO animals (data not shown). Study of more prolonged PA infection may reveal a role for IL-22 in preventing aberrant lung repair and fibrosis.

Although interleukin-22 has attracted attention as a critical mediator of mucosal immunity, we found no evidence that it influences the short or longer-term outcome in experimental chronic pulmonary *P. aeruginosa* infection. Critical to therapeutic targeting and CF pathogenesis, IL-22 failed to protect against the development of *P. aeruginosa* colonization. However, we identified a novel role for IL-22 in exacerbating infection-induced weight loss, an important prognostic factor in patients with cystic fibrosis, which warrants further investigation.

The following are the supplementary data related to this article.

Supplementary Table 1 Characteristics of CF patients undergoing lung transplantation where airway lavage fluid +/- explanted lung tissue was obtained. Supplementary Table 1.

Supplementary Fig. 1Levels of BAL and lung homogenate cytokines in mice treated with sterile beads and *Pseudomonas aeruginosa-*laden beads.C57Bl6 mice were treated with intrapulmonary sterile agar beads or agar beads laden with *P. aeruginosa* strain NH57388A. BAL IL-17F (a), BAL IL-21 (b) and lung homogenate IL-17A levels measured at 2 weeks post-instillation of sterile agar beads or beads laden with NH57388A. Line represents median. *P*-values denote comparison by Mann–Whitney test. , animals with ongoing pulmonary PA infection.Image 1

## Disclosures

None.
