# Supplementary material for: IL-22 exacerbates weight loss in a murine model of chronic pulmonary Pseudomonas aeruginosa infection
Source: J Cyst Fibros. 2016 Nov;15(6):759–68. doi: 10.1016/j.jcf.2016.06.008 (PMC5154339; doi:10.1016/j.jcf.2016.06.008)
Supplement: Supplementary Table 1 — Characteristics of CF patients undergoing lung transplantation where airway lavage fluid +/- explanted lung tissue was obtained. [file mmc1.docx]

| **Age**  **(years)** | **Cystic Fibrosis Transmembrane Conductance Regulator**  **Genotype**  **(where known)** | **Forced Expiratory Volume In 1 Second**  **(% predicted)** | **Peri-operative Sputum Microbiology** |
| --- | --- | --- | --- |
| 20 | F508del / F508del | 21 | P. aeruginosa |
| 21 | Unknown | 25 | P. aeruginosa |
| 22 | F508del / F508del | 23 | P. aeruginosa |
| 23 | Unknown | 27 | P. aeruginosa, A. fumigatus, A. xylosoxidans, M. avium complex |
| 24 | F508del / F508del | 26 | P. aeruginosa |
| 25 | F508del / Unknown | 22 | *Pseudomonas aeruginosa, Staphylococcus aureus* |
| 28 | Unknown | 20 | P. aeruginosa, Methicillin-Resistant S. aureus |
| 29 | F508 del / 2184insA | 17 | Burkholderia multivorans, Aspergillus fumigatus, Geosmithia argillacae |
| 29 | F508del / F508del | 19 | P. aeruginosa, Stenotrophomonas maltophilia, Achromobacter xylosoxidans, A. fumigatus, G. argillacae, Mycobacterium avium complex |
| 39 | F508del / F508del | 14 | P. aeruginosa |
| 40 | Unknown | 23 | P. aeruginosa |
| 45 | F508del / F508del | 21 | P. aeruginosa, A. fumigatus |
| 46 | Unknown | 30 | P. aeruginosa |
| 48 | F508del / Unknown | 29 | P. aeruginosa, S. aureus |

**Supplementary Table 1** – Characteristics of CF patients undergoing lung transplantation where airway lavage fluid +/- explanted lung tissue was obtained
