# Supplementary material for: IL-22 exacerbates weight loss in a murine model of chronic pulmonary Pseudomonas aeruginosa infection
Source: J Cyst Fibros. 2016 Nov;15(6):759–68. doi: 10.1016/j.jcf.2016.06.008 (PMC5154339; doi:10.1016/j.jcf.2016.06.008)
Supplement: Supplementary Fig. 1 — Levels of BAL and lung homogenate cytokines in mice treated with sterile beads and Pseudomonas aeruginosa-laden beads. C57Bl6 mice were treated with intrapulmonary sterile agar beads or agar beads laden with P. aeruginosa strain NH57388A. BAL IL-17F (a), BAL IL-21 (b) and lung homogenate IL-17A levels measured at 2 weeks post-instillation of sterile agar beads or beads laden with NH57388A. Line represents median. P-values denote comparison by Mann–Whitney test. Image 2, animals with ongoing pulmonary PA infection. [file mmc2.pptx]

## Slide 1
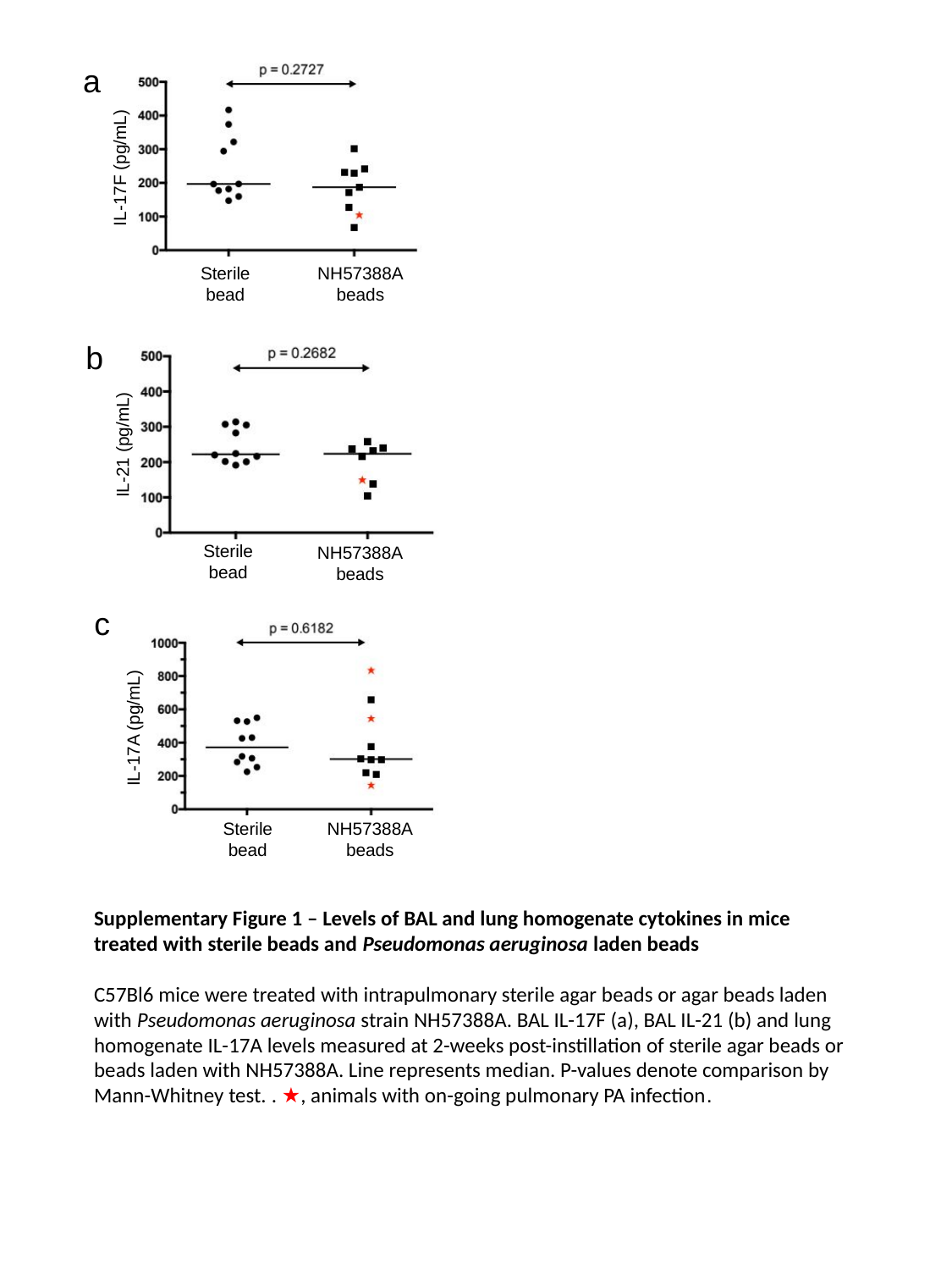

a
IL-17F (pg/mL)
Sterile
bead
NH57388A
beads
b
IL-21 (pg/mL)
Sterile
bead
NH57388A
beads
c
IL-17A (pg/mL)
NH57388A
beads
Sterile
bead
Supplementary Figure 1 – Levels of BAL and lung homogenate cytokines in mice treated with sterile beads and Pseudomonas aeruginosa laden beads
C57Bl6 mice were treated with intrapulmonary sterile agar beads or agar beads laden with Pseudomonas aeruginosa strain NH57388A. BAL IL-17F (a), BAL IL-21 (b) and lung homogenate IL-17A levels measured at 2-weeks post-instillation of sterile agar beads or beads laden with NH57388A. Line represents median. P-values denote comparison by Mann-Whitney test. . ★, animals with on-going pulmonary PA infection.
80
